# From observation to evidence: harnessing the mental status examination as a real-world data source in clinical research

**DOI:** 10.3389/frhs.2026.1784842

**Published:** 2026-07-23

**Authors:** Mayowa Oyesanya, Christoph U. Correll

**Affiliations:** 1 Holmusk Technologies Inc, New York, NY, United States; 2 Department of Child and Adolescent Psychiatry, Charité - Universitätsmedizin Berlin, Berlin, Germany; 3 German Center for Mental Health (DZPG), Berlin, Germany; 4 Department of Psychiatry and Molecular Medicine, Donald and Barbara Zucker School of Medicine at Hofstra/Northwell, Hempstead, NY, United States

**Keywords:** electronic health record, large language models, measurement-based care, mental status examination, natural language processing, psychopathology, real-world evidence, schizophrenia

## Abstract

**Background:**

Electronic health record (EHR) based research using psychiatric real-world data is limited by a lack of measurement-based care. The mental status examination (MSE) offers a routinely documented, alternative data source for clinical phenotyping, potentially addressing this evidence gap.

**Aim:**

This article aims to provide a comprehensive clinical and scientific justification for the use of MSE data as a source of real-world evidence, and to critically examine the assumptions underlying its use.

**Discussion:**

The content and structure of the MSE are first described and compared with those of structured psychometric rating instruments. Current literature applying natural language processing (NLP) methods, (including large language models (LLMs)), to extract psychopathological information from the EHR is briefly reviewed. Schizophrenia is then used as a case study for an in-depth critical examination of the assumptions and complexities inherent in mapping MSE findings to their corresponding psychopathological constructs. Principles for subject-matter-expert review of MSE data are proposed alongside a sample conceptual organization of MSE findings according to the main psychopathological domains of schizophrenia. Contributions made by MSE data at the research frontier are discussed, and the limitations of MSE data are considered.

## 1 Introduction

Contemporary psychiatry operates in a substantially different environment from the one Karl Jaspers knew (1). As the father of descriptive psychopathology, he aimed to systematically describe rather than explain psychopathology. This approach remains in modern psychiatric practice, though in contrast to Jaspers' time, psychiatrists generally use computers to document their findings. This digital transition has facilitated the use of electronic health records (EHRs) for research, but significant challenges persist.

Research using real-world data has been constrained by the incomplete implementation of measurement-based care leading to the variable presence of psychometric instruments in the EHR (2). Mental status examination (MSE) data can potentially address this gap by providing a complementary and routinely documented clinical data source that serves as the primary locus of clinically observed psychopathology in the clinical record. However, the potential utility of MSE data is constrained by the realities of clinical practice. Documented MSEs can vary in their quality and comprehensiveness, and the subjective nature of psychopathological assessment can impact interrater reliability (3). Using MSE data also does not address the labor-intensive nature of information extraction from unstructured real-world data.

Deep learning natural language processing (NLP) technologies enable the efficient extraction of relevant data from unstructured or semi-structured sources. Numerous studies have used NLP techniques to extract or organize symptom data from EHRs focusing either on specific psychiatric disorders or the extraction of transdiagnostic symptoms and signs (4–10). Fewer studies have focused explicitly on extracting data from the MSE, and schizophrenia patients were commonly studied (4, 5, 6, 11, 12). These studies have demonstrated how MSE data can be used to identify psychiatric disorder symptoms and signs and adverse outcomes such as increased healthcare resource utilization and costs in naturalistic settings (11, 12).

Despite advancements in the use of NLP techniques on EHR data, the inherent complexities of the MSE still necessitate its review by subject matter experts. Such expert oversight is not only required for the accurate clinical interpretation of MSE data, but for its subsequent conceptual organization. This conceptual organization is necessary to systematically organize MSE findings according to equivalent, formal descriptions of psychopathology.

The literature currently lacks an extensive critical analysis of the assumptions underpinning the conceptual organization and application of real-world MSE data for identifying psychopathology. This article seeks to critically evaluate both the MSE as a data source, and the assumptions inherent to its conceptual organization. Schizophrenia will be used as the main psychiatric disorder to provide context for this discussion.

## 2 Background

### 2.1 The mental status examination: definition, structure, and related clinical concepts

The MSE is the direct conceptual equivalent in psychiatry to the physical examination in other medical specialties. It formalizes the systematic description of patient psychopathology, aiming to identify abnormal cognitions, emotions, and behaviors indicative of mental illness (13, 14). There are two broad types of finding from the MSE: signs which reflect what the examiner observes, and symptoms which reflect what the patient reports. The MSE is predominantly composed of signs rather than symptoms with patient reported mood, thought content, and perception being important exceptions. In this article, MSE “findings” will be used as an umbrella term for psychopathological signs and symptoms that are documented in the MSE itself.

The MSE utilizes a clinical structure and nomenclature, ensuring that all relevant “systems” are assessed and that findings are readily interpretable by other clinicians who have not personally assessed the patient. The MSE is structured into domains within which specific examination findings are reported. These domains relate to different areas of assessment for psychopathology. Table 1 in the section provides an overview of the core domains of the MSE.

**Table 1:** Core domains of the mental status examination with explanations.

MSE Domain	Explanation
Appearance	Clothes worn and their appropriateness, level of hygiene/grooming, general physical condition
Behavior	Motor activity during the interview; abnormal movements.
Affect	Emotional expression as manifested by facial expression and other observable markers
Mood	Sustained emotional state.
Speech	Rate, rhythm, flow, intonation, and prosody.
Thought Process	Flow, coherence, logicality, and organization of thinking as inferred via speech
Thought Content	Self-reported thoughts (e.g., delusions, suicidal ideation).
Cognition	Memory, attention, orientation, etc.
Insight	The degree to which a patient is aware of their mental health and that they may be suffering from a psychological abnormality

Documented MSE data may directly or indirectly refer to psychopathological constructs. For the purposes of this article, a psychopathological construct is a precise formal designation for a clinically identifiable phenomenon whose presence evidences a mental disorder.

### 2.2 Comparing the MSE with psychometric rating instruments

Structured rating instruments are a vital data source in electronic health records and have specific properties that are useful to explore in comparison with MSE data. Rating instruments are *measurement* tools that describe both the presence and extent of a predefined number of psychopathological constructs. In contrast, the MSE is a clinical examination which reflects *observations* made by an assessing clinician or information obtained during a clinical encounter. The MSE is inherently cross-sectional, as it reflects observations at the time of assessment, while rating instruments can be cross-sectional or longitudinal in their assessment scope. The MSE is inherently diagnosis-agnostic, whereas rating instruments in psychiatry are frequently designed to capture signs and symptoms specific to a particular disorder. However, more transdiagnostic rating instruments are being developed in psychiatry (15–17).

The MSE can often provide more phenotypic specificity than rating instruments. For instance, the Positive and Negative Syndrome Scale (PANSS) (18), the gold standard rating instrument for assessing response to psychiatric treatment in schizophrenia, assesses “conceptual disorganization” (disorganized thinking) within a single item. In contrast, this psychopathological construct may be described in different ways in the MSE e.g., “circumstantiality” or “loosening of associations” These descriptors provide more clarification about the nature and degree of disorganized thinking. While the MSE can provide increased detail, this must be weighed against the possibility of incomplete or insufficient documentation of MSE findings. Naturally, a more comprehensive MSE will take more time and effort to complete, which may limit the depth of MSEs documented by time-pressured clinicians.

Beyond specificity, MSE and rating instrument data can differ in their completeness of coverage. Documented MSE findings likely reflect clinically salient signs or symptoms, rather than an exhaustive description of all observable psychopathology (19). For clinical problems with a special significance, such as psychosis or suicidality, competent clinicians are unlikely to omit relevant details, given the significant medicolegal risks associated with inaccurate or incomplete documentation of these clinical presentations. On the other hand, as rating instruments are standardized and designed to be completed in full, a properly completed rating instrument does not suffer from this *salience effect*, and this is a key relative strength of rating instrument data in research. It should be noted that interrater agreement for the assignation of diagnoses according to the classification of the 5th edition of the Diagnostic and Statistical Manual of Mental Disorders (DSM-5) is only fair to moderate (Cohen's kappa of 0.4–0.6) (20). This partly reflects the interrater reliability of the MSE that formed the basis for the diagnosis, as raters were trained on the DSM-5 criteria prior to performing an independent examination.

To address these variations in diagnostic and assessment reliability, postgraduate curricula for psychiatrists in training (residents) aim to standardize the ways in which they identify and document psychopathology. For instance, organizations such as the US Accreditation Council for Graduate Medical Education (ACGME) mandate specific training frameworks to ensure residents achieve the core competencies required for robust clinical assessment (21). This standardization is operationalized through a combination of direct clinical observation (22), standardized examinations, and national performance benchmarking (23, 24). While these structured milestones provide quality control for psychiatric residents, other health professionals performing the MSE may lack similarly regulated and monitored training in psychopathological assessment. This variability in training could potentially compromise the quality and consistency of conducted mental status examinations, particularly outside specialist psychiatric settings.

## 3 Overview of schizophrenia symptoms

Schizophrenia provides a useful case study for examining how the properties of MSE data apply in practice given the well characterized and distinct domains of the disorder. These domains include positive, negative, and cognitive symptoms which are well defined in diagnostic manuals and the academic literature (25). The DSM-5 and the International Classification of Diseases 11th edition (ICD-11) provide the firmest consensus for essential schizophrenia signs and symptoms to support diagnosis. They describe core signs and symptoms similarly though they are organized differently (26–28).

Positive symptoms reflect excessive or abnormal cognitions, emotions, or behaviors. Specific positive symptoms include hallucinations (a sensory experience in the absence of an objective stimulus), delusions (a fixed often false belief that is resistant to contrary evidence), conceptual disorganization (disorganized thought processes as manifested in speech), and abnormal or disorganized behaviors (29).

Negative symptoms reflect a diminishment or abnormal absence of particular functions, behaviors and experiences (30). Clinical research and consensus support the organization of negative symptoms into five distinct psychopathological constructs, which are underpinned by an underlying expressive symptom factor and a motivational/experiential symptom factor (30). These five psychopathological constructs are: alogia (diminishment in the quality and volume of speech), anhedonia (reduction or absence in the ability to experience pleasure), avolition (a reduction in goal directed behavior), social amotivation/asociality (reduced or absent social drive), and diminished emotional expression (30). The DSM-5 dedicates a section to describing negative symptomatology in schizophrenia, ascribing particular prominence to avolition and diminished emotional expression, while also listing, alogia, asociality, and anhedonia (30–32).

While cognitive impairment associated with schizophrenia (CIAS) is recognized in both the DSM-5 and ICD-11, it is most comprehensively operationalized within the Measurement and Treatment Research to Improve Cognition in Schizophrenia (MATRICS) Consensus Cognitive Battery (MCCB) (33). Regarded as the gold standard for formal cognitive assessment in schizophrenia, the MCCB captures several distinct CIAS domains that represent fundamental and distinct cognitive domains of the illness: processing speed, attention, working memory, verbal learning and memory, visual learning and memory, problem solving/reasoning, and social cognition (34, 35).

### 3.1 Clinical considerations in the conceptual organization of MSE data in schizophrenia patients

Positive, negative, and cognitive signs and symptoms can be identified in the MSE data of schizophrenia patients, but these psychopathological domains can vary in how directly they are represented in MSE documentation. Conceptual organization of MSE data is therefore interpretive and judgment-based, requiring alignment of MSE findings with their equivalent psychopathological constructs. The greater this divergence, the more interpretive judgment is required for conceptual organization.

The manifestations of psychopathological constructs in schizophrenia may be described in the MSE, providing indirect evidence for the presence of these constructs. A patient may be described as having “poor hygiene” or “poor self-care” which can be a downstream manifestation of avolition; indeed, several rating instruments use this clinical sign as an avolition proxy (36–38).

Similarly, CIAS is rarely formally assessed in real-world clinical practice, meaning that downstream manifestations are more likely to be described. These could include: documented socially inappropriate speech or behaviors (social cognition impairment); impairments in patient judgment (reasoning and problem solving impairment); difficulties with following the flow of a conversation (processing speed/attention problems); or poor performance in bedside cognitive tests such as the reverse digit span (working memory).

Some schizophrenia symptoms can be more easily identified on examination reflecting their more obvious clinical manifestations. Positive symptoms are usually readily evident in an examination context and tend to be clearly described. Hallucinations and delusions tend to be documented by their sensory modality (e.g., auditory hallucinations) or precise form (e.g., grandiose delusions); though indirect descriptions can also be used e.g., a patient “openly responding to unseen stimuli” as an indicator of hallucinations. Abnormal behaviors and disorganized thinking are similarly apparent from direct observation of behaviors and speech respectively. These psychopathological constructs are documented within clearly demarcated MSE domains: Perception (hallucinations and other altered perceptions), Thought Content (Delusions), Thought form (disorganized thinking), and Behavior.

Experiential negative symptoms, such as anhedonia, avolition and social amotivation, which require direct questioning and observation to properly identify, may be reported less effectively than expressive symptoms which tend to be more apparent (alogia and diminished emotional expression are commonly documented as “poverty of speech” and “blunted affect” respectively).

Documented examination findings may plausibly map to several psychopathological constructs. A schizophrenia patient reported as “disengaged” may reflect a reduced drive for social interaction in the encounter (social amotivation). However, perceived disengagement may also be a consequence of a reduced ability to initiate or persist with goal directed behaviors (avolition), specifically the verbal and nonverbal behaviors that would demonstrate social engagement. These ambiguities can complicate conceptual organization and provide a justification for organizing symptoms more broadly, e.g., within a broader motivational-experiential negative symptom dimension.

### 3.2 Principles for review of MSE data by subject matter experts

Review of an MSE data corpus by subject matter experts (ideally specialist mental health clinicians) can guide its conceptual organization. The principles outlined in this section apply regardless of MSE format.

#### 3.2.1 Principle 1: operationally defining psychopathological constructs

Operationally defining the psychopathological constructs or groupings that MSE data will be mapped to can be challenging. There can be multiple plausible but potentially discrepant operational definitions. This problem can be addressed by prioritizing sources that represent a strong consensus for the definition of psychopathological constructs. For schizophrenia this could reasonably be the ICD-11 or DSM-5 which are largely and intentionally harmonized in their description of core psychopathological constructs (39, 40). For cognitive symptoms which are not core diagnostic criteria for schizophrenia in either manual, the MCCB is the definitive source (33).

#### 3.2.2 Principle 2: distinguishing psychopathological constructs, psychiatric diagnoses, and MSE findings

A clear understanding of three distinct levels of description is necessary for organizing MSE data: the formal psychopathological construct, (e.g., alogia) its description in reference sources (e.g., DSM-5), and the MSE findings that are indicative of it (e.g., impoverished speech). Failing to explicitly delineate these descriptive levels precludes the development of coherent, theoretically grounded principles for conceptually organizing MSE data.

#### 3.2.3 Principle 3: understanding different relationships between an MSE finding and a psychopathological construct

The third principle is an appreciation of the different conceptual levels at which MSE findings can be mapped to psychopathological constructs. These different conceptual levels include but are not necessarily limited to:

(1) Direct or verbatim descriptions of a construct, where the documented finding explicitly names the construct itself (e.g., documenting “alogia” or “anhedonia”).
(2) Synonymous descriptions of a psychopathological construct, e.g., documenting “impoverished speech” instead of “alogia”.
(3) Downstream manifestations or correlates of a psychopathological construct. These MSE findings are *clinically inferred* to reflect an underlying construct, and their identification is conditional and context-dependent. As previously discussed, the finding of poor hygiene can reasonably be considered a manifestation of avolition in schizophrenia and therefore an indicator of psychopathology. It would be less reasonable to consider poor hygiene as such an indicator in an individual with no psychiatric diagnosis and no other discernible psychopathology.

The delineation outlined in this section matters because each level of description requires increasing clinical interpretation and subjective judgment. Direct or synonymous descriptions have a firmer *evidentiary status* than downstream descriptions of psychopathological constructs. However, distinctions between synonymous representations of psychopathological constructs and downstream manifestations may be a matter of judgment in some cases.

#### 3.2.4 Principle 4: establishing interrater reliability

The fourth and final principle concerns the empirical reliability of the mapping process itself; it is important to formally assess the interrater reliability of any such conceptual organization effort using standard methodological means. The conceptual scheme for mapping MSE findings should ideally be pre-specified. Distinguishing between direct, synonymous, and correlative evidence of psychopathology is helpful, although it is likely that this additional complexity will reduce interrater reliability.

Table 2 provides a simplified schematic demonstrating the conceptual organization of MSE findings in a schizophrenia patient according to recognized psychopathological constructs or groupings.

**Table 2:** Sample MSE findings mapped to the positive, negative, and cognitive symptom domains of schizophrenia.

Schizophrenia symptom supercategory	Schizophrenia symptom subcategory	Sample MSE Examination Finding
Positive symptoms	Hallucinations	Auditory hallucinations, visual hallucinations, tactile hallucinations
	Conceptual disorganization	Non-linear thinking, disorganized thinking, illogical thinking, incoherent thinking, loosening of associations, formal thought disorder
	Delusions	Persecutory delusions, paranoid delusions, delusions of grandeur, delusions of reference
	Disorganized or abnormal behaviors	Posturing, catatonic, negativism, gesturing, stereotypies
Negative symptoms	Diminished expression (alogia, affective blunting)	Blunted affect, monotone speech, speech latency, impoverished speech
	Diminished motivation and experiences (avolition, anhedonia, social amotivation)	Apathetic, withdrawn, uninterested, disengaged, reports anhedonia
Cognitive symptoms	Memory	Reduced word recall (bedside test of memory), reduced reverse digit span (indicative of working memory impairment),
	Attention	Unable to focus, impaired attention, inattentive, distracted, distractible
	Executive functioning	Impaired decision making, impaired judgment, poor understanding of safety
	Processing speed	Slow to process [information] (assessment of processing speed is uncommon in the MSE)
	Social cognition	Hostile, aggressive, inappropriate remarks, bizarre dress/appearance

Examination findings are illustrative and intend to represent possible clinical documentations assuming that each symptom domain was assessed. The DSM-5 was used as the conceptual scheme for negative and positive symptoms. The MATRICS Consensus Cognitive Battery was used as the conceptual scheme for cognitive symptoms. This scheme is not intended to be a comprehensive and exhaustive documentation of the array of clinical terms used to describe signs and symptoms.

## 4 Research at the frontier: cognitive impairment in schizophrenia

Identifying CIAS in real-world data has historically been limited by a lack of structured assessment in real world clinical practice and general under-recognition. It therefore serves as a good example of how newer research techniques may enable larger scale analysis of real-world data including MSE data. NLP techniques have helped to address this problem by enabling the efficient extraction of unstructured documentations of CIAS in clinical notes. Recently published evidence using NLP techniques on EHR data identified CIAS in 25.2% of patients (41). This estimate is lower than recently published or presented estimates derived from: a combination of MSE, rating instrument, and NLP-derived data (59%) (42), or medical expenditure survey data (53.8% for cognitive difficulties and 57.7% for cognitive limitations) (43). Previous literature in this area suggests that CIAS affects approximately 80% of patients (44, 45).

CIAS identified within MSE data has been shown to independently predict psychiatric hospitalization (46) (Hazard Ratio (HR) = 1.23, 95% CI = 1.16–1.31, *P* < 0.001), aligning with the present literature (11, 47). This retrospective cohort analysis was performed with Cox and general linear models, and adjusted for potential confounders: substance use disorder diagnoses, antipsychotic prescriptions and illness severity. This approach provides preliminary evidence that MSE can be predictively validated, reproducing known associations between cognitive impairment and clinical outcomes. Caution must of course be taken in interpreting prevalence and effect estimates derived from heterogeneous data sources and populations. These data were presented at a scientific conference and have not yet been published in a peer-reviewed journal. However, overall, these findings demonstrate that MSE data are making useful contributions to an area of research where published research remains sparse.

## 5 The limitations of MSE data

The following contextual factors may affect the usefulness and interpretability of MSE data used for research: the clinical setting, clinician seniority and professional background, clinical specialty, clinical acuity, temporal constraints, and diagnostic suspicion. Variability in these factors can systematically influence the descriptive granularity, comparability, reliability, volume and longitudinal availability of MSE data.

In making judgments about the usefulness or appropriateness of MSE data for a particular research indication, this variability should temper the confidence placed in affected findings and the generalizability of the data. Researchers should consider the provenance of MSE data, and data from specialist settings may yield higher-quality data for identifying specific psychopathological constructs. It must also be acknowledged that the level of clinical understanding and judgment required to make optimal use of MSE data, may create an entry barrier for researchers lacking this knowledge. This can limit the ease of use of MSE data compared with rating instrument data.

The clinical heterogeneity of MSE data may limit or complicate its longitudinal analysis. If, for example, the organizing structure of documented MSEs changes substantially across time, then a sequence of MSEs may not be directly comparable. A change in documented findings also does not necessarily reflect a genuine change in the corresponding psychopathology; it may instead reflect changes in documentation and assessment practices.

The MSE does not provide comprehensive data about all features of psychopathology, especially those that need to be measured precisely (e.g., specific cognitive impairments) or those that may require longitudinal assessment (e.g., social amotivation). As the MSE is based on clinical observation and interview, it is possible that important aspects of the subjective experience are missed, misinterpreted, under-recorded or de-emphasized due to the assessor's approach, or the patient being uncooperative or mentally unwell. Taken together, these limitations do not preclude the use of MSE data for research, but they demonstrate the importance of critically appraising data provenance, and applying due caution in the interpretation of findings.

## 6 Conclusions

This article has provided a perspective about current uses for MSE data, and the underlying assumptions and complexities associated with its conceptual organization. Clinical expertise and judgment are essential to guide the review, interpretation, and decision to use MSE data for research. An intriguing research area which logically follows from arguments established in this article, is the assessment of whether NLP technologies guided by a ground-truth corpus of conceptually organized MSE data, can automatically extract and distinguish between direct and indirect descriptions of psychopathology, with strong classification performance. One imagines that if Jaspers were alive today, he would be excited by this prospect.

## Data Availability

The original contributions presented in the study are included in the article, further inquiries can be directed to the corresponding author/s.
